# Effective Properties for the Design of Basalt Particulate–Polymer Composites

**DOI:** 10.3390/polym15204125

**Published:** 2023-10-18

**Authors:** Jong-Hwan Yun, Yu-Jae Jeon, Min-Soo Kang

**Affiliations:** 1 Mobility Materials-Parts-Equipment Center, Kongju National University, Gongju-si 32588, Republic of Korea; yunjh0915@kongju.ac.kr; 2 Department of Medical Rehabilitation Science, Yeoju Institute of Technology, Yeoju 12652, Republic of Korea; superlittle@yit.ac.kr; 3 Division of Smart Automotive Engineering, Sun Moon University, Asan-si 31460, Republic of Korea

**Keywords:** polymer, composite, basalt powder

## Abstract

In this study, preliminary simulations were performed to manufacture thermoplastic composites that can be processed by injection. For analysis, a basalt particulate–polymer composite model was manufactured and its elastic modulus, shear modulus, thermal expansion coefficient, and thermal conductivity were predicted using finite-element analysis (FEA) and micromechanics. Polypropylene (PP), polyamide 6, polyamide 66, and polyamide (PA) were employed as the polymer matrix, with the variations in their properties investigated based on the volume fraction of basalt. The polymer–basalt composite’s properties were analyzed effectively using FEA and the micromechanics model. FEA was performed by constructing a 3D model based on the homogenization technique to analyze the effective properties. The micromechanics model was analyzed numerically using the mixture rule, and the Mital, Guth, and Halpin–Tsai models. As a result, it is best to analyze the effective properties of polymer–basalt composites using the Halpin–Tsai model, and it is necessary to conduct a comparative analysis through actual experiments. In the future, actual composite materials need to be developed and evaluated based on the findings of this study.

## 1. Introduction

The automotive industry is undergoing significant changes because of the effects of climate change, leading to automobile makers focusing on reducing pollutants and improving fuel efficiency in vehicles powered by traditional internal combustion engines. Furthermore, with the development of electric vehicles, lightweight components have become essential in terms of fuel efficiency and range in the automotive sector. Therefore, the automotive sector requires technology that enables lighter vehicles while maintaining safety, performance, and durability [1]. According to previous research, a 10% reduction in vehicle weight results in a 7% increase in fuel efficiency [2], because 50% of fuel consumption is determined by vehicle weight [3]. Generally, reducing a vehicle’s weight by 100 kg reduces CO_2_ emissions by 20 g/km [4]. Hence, utilizing and developing lightweight materials in the automotive sector is a key task. Various research attempts have been made to fabricate lightweight car materials. The most common lightweight materials for cars are polymer-based composites.

Three reinforcement mechanisms can be used for composites. The first is the particle-reinforcement mechanism, which generates a composite by dispersing particles inside the base material, displaying the isotropic shape of the dispersed phase of the reinforcement. The second is the fiber-reinforcement mechanism, in which the reinforcing materials are typically in the form of fibers [5,6,7], either continuous or short-interrupted. The last reinforcement mechanism is the structural composite, formed by the structural combination of pure components. Sandwich panels and laminar composites are examples of structural composites. Polymer composites are created and used in industries based on the formation mechanism of these composites. To broaden the spectrum of usage of composite materials, injection-molding processes for composite resins must be available to produce composite materials for various parts utilized in industry. In the case of composites with fiber-reinforcement mechanisms, injection molding is impractical and a high-temperature molding process must be used, making mass production of composite components difficult and expensive. Therefore, creating and using particle-reinforced composite materials that allow effective injection molding is critical. However, injection molding is a complicated process because the polymer composite must encounter inhomogeneous shear flow in a relatively short time during injection molding [8,9]. Composites are further classified into thermosetting and thermoplastic composites based on the type of polymer utilized. Owing to their ease of manufacture and simple molding process, thermoset composites have accounted for most of the polymer composites used in the automotive industry. However, owing to recycling challenges, they are becoming increasingly regulated; for instance, the European Union’s end-of-life vehicle (ELV) directive requires manufacturers to reuse and recover at least 95% of the weight per vehicle [10,11].

Hence, in the automobile industry, thermoset composites are rapidly being replaced with thermoplastic composites. Thermoplastics include polyethylene, polypropylene (PP), nylon, polyvinyl chloride, polyvinyl acetate, polystyrene, ABS (acrylonitrile butadiene styrene copolymer), and acrylic resins. Among these thermoplastics, PP is a thermoplastic polymer with excellent economic, biological, physical, chemical, and thermal properties. PP polymer has low density, is semi-rigid, odorless, and tough, and has good chemical and fatigue resistance and long life; PP is also resistant to fungi, bacteria, effective acids, and bases and has low moisture retention. Pipes, plates, films, chairs, electrical materials, automotive parts, rope, and yarn are used as basic materials in the manufacturing of many polymer composites [12,13,14]. Furthermore, nylon resin is a high-performance material with exceptional hardness, heat resistance, and oil resistance and is widely utilized for various commodities, packaging, and building materials, particularly in electronic and mechanical components such as vehicle parts and connections. When polyamide (PA) is present in the form of a line, these nylon resins are called nylon. Although various types of PA exist, PA6 and PA66 are those most often utilized. PA6 offers exceptional low-temperature toughness as well as outstanding damping and impact resistance. It is also a high-performance material with good wear resistance when bonding pieces on rough surfaces. As a result, PA66 is frequently used to replace metal parts, owing to its high mechanical strength. Various additives are blended inside this basic material as a matrix to generate composite materials used in various industries.

Among the most recent reinforcements, we are developing a polymer-reinforcing mechanism employing basalt. Basalt is a noncombustible material with heat dissipation, heat resistance, sound insulation, sound absorption, dust resistance, erosion resistance to acids and alkalis, corrosion resistance to water, abrasion resistance, and lightweight high-strength properties. Most studies on basalt composites have focused on composites with fiber-reinforced mechanisms [15,16,17,18]. Conducting these simulation studies requires a quantitative data analysis of each matrix material [19]. In addition, the existing studies are basalt fiber-centric property prediction studies, and the micromechanics model used is basically the Mori–Tanaka method [20,21]. Consequently, simulations are required to study the effective properties of composites to produce composites comprising polymer and basalt powders that can be processed by injection molding. In such simulation studies, it is necessary to homogenize the composite material before producing test samples and simulating a physical and mechanical analysis on these samples. Only in this way can the structural mechanical properties be accurately predicted. Mean field homogenization methods are used at the microscale level to obtain optimal mechanical properties, such as the elastic modulus, of matrix materials with nano/microscale filler materials. After this level, mesoscale methods are used that take into account fibers/particles or reinforcing matrices, with which a homogeneous material model is achieved at the structural level [22,23]. Ansys Material Designer allows researchers to estimate composite water using preloaded geometric definitions of RVEs and boundary conditions [24]. Currently, most of the research using basalt is based on fiber composites. There is a lack of research on powder-based composites for isotropic composite manufacturing and injectionable materials.

Thus, in this study, PP, PA6, PA66, and PA were chosen as the matrixes, with basalt powder used as the reinforcement to create an injection-molded isotropic composite material. The effective properties of composites with a mixed matrix and reinforcement were investigated using finite-element analysis (FEA) and the Ansys micromechanics model. The effective properties were evaluated and studied according to the volume fraction of the additive basalt particulate and polymer matrixes.

## 2. Materials and Methods

**Figure 1:**
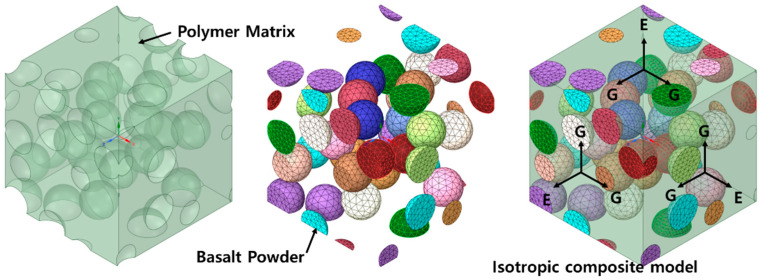
Finite-element analysis model (model images according to volume fraction).

In this study, the homogenization technique was used in ANSYS Material Designer to assess the effective properties of composites made of thermoplastic polymer and basalt powders. For three-dimensional (3D) composites, the establishment of a representative volume element (RVE) under periodic boundary conditions based on the homogenization principle and finite-element method can predict the mechanical properties and damage mechanism of the composites [25,26]. A 3D model of a composite with a powder combination was used to replicate the composite model described in this study. All planes had symmetrical boundary conditions in FEA via homogenization theory. As a result, all sides of the RVE were subjected to symmetric boundary conditions. Figure 1 depicts the composite-material model for FEA, which indicates the direction of each force. The volume in the analytical model is set to Matrix (PP, PA6, PA66, PA). Figure 1 shows the 3D fundamental model of the particle composite as well as the tensors employed. E represents the elastic modulus, and 1, 2, and 3 represent the x, y, and z axes, respectively. Furthermore, G represents the shear stress created on the composite face. Tensors are represented by the subscripts m for matrix and p for internal additive.

The basalt powder was arranged randomly inside the RVE. In addition, to construct the 3D-analysis model, the volume proportion of basalt powder in the entire composite model was set from 0 to 35. The mesh type utilized in the FEA was SOLID187. The SOLID187 element has ten element nodes and is characterized by a secondary displacement mode and relatively high computational accuracy [27]. Moreover, the element dimension was set at 0.5 m. SOLID187 is well suited for creating meshes inside irregularly shaped structures because it supports second-order displacement [28]. Moreover, the element sizes in the matrix and basalt powder were fixed to the same condition to reduce the sensitivity of the effective property prediction of the FEA to the mesh size. Table 1 shows the physical parameters of the materials employed in this study.

Many micromechanical models have been created to study the effective properties of composite materials. The fibers, matrix, and, sometimes, the interface are assumed to be continuous materials in these micromechanical models, with the constitutive equations for the bulk composite material formulated using continuous mechanics assumptions [35,36]. Micromechanical models were utilized in this study to estimate the theoretical elastic properties of composite materials and were compared to the findings of the FEA. The rule of mixture (ROM) is the most commonly used method for estimating the effective properties of materials in mixed-form composites. It provides theoretical upper and lower bounds on properties such as the elastic modulus, ultimate tensile strength, thermal conductivity, and electrical conductivity [37]. In general, two types of ROM are used: the Voigt model, used to examine axial loading, and the Rouss model, used to study transverse stress. The ROM employed in this study was estimated using the Voigt model and the powder–material composite properties. Equations (1)–(4) are micromechanics equations using the ROMs for the elastic modulus (E), Poisson’s ratio (ν), thermal-expansion coefficient (α), and thermal conductivity (κ) [38,39]. The properties of each material featured in the ROM model were shown to be linearly dependent on the matrix and particle-volume fraction.

(1) $$E_{C\_ROM}=E_{m}V_{m}+E_{p}V_{p}$$

(2) $$G_{C\_ROM}=G_{m}V_{m}+G_{p}V_{p}$$

(3) $$\alpha_{C\_ROM}=\alpha_{m}V_{m}+\alpha_{p}V_{p}$$

(4) $$\kappa_{C\_ROM}=\kappa_{m}V_{m}+\kappa_{p}V_{p}$$

Mital and Glodberg [40,41] established a micromechanics model to describe the micromechanical equations for particle-reinforced composites and estimate the effective thermal and mechanical properties arising from applied thermal and mechanical microstresses. Particles of varied shapes and sizes are uniformly dispersed in a binder mate-rial for particle-reinforced composites. The particulate material is assumed to be uniformly distributed in a cubic lattice as spherical particles with a diameter that is the mean value of a range of diameters in composites, where the particles are assumed to be arranged in a regular arrangement pattern such as square or hexagonal. Furthermore, the distance between nearby particles is also assumed to be calculable using the total particle volume fraction. The micromechanics model equations derived by Mital are as follows:

(5) $$E_{C\_Mital}=\frac{{V_{p}}^{0.67}E_{m}}{1-{V_{p}}^{0.33}(1-\frac{E_{m}}{E_{p}})}+(1-{V_{p}}^{0.67})E_{m}$$

(6) $$G_{C\_Mital}=\frac{{V_{p}}^{0.67}G_{m}}{1-{V_{p}}^{0.33}(1-\frac{G_{m}}{G_{p}})}+(1-{V_{p}}^{0.67})G_{m}$$

(7) $$\alpha_{C\_Mital}=\tilde{\alpha }{V_{p}}^{0.67}\frac{{\tilde{E}}_{p}}{E_{C\_Mital}}+\alpha_{m}\frac{E_{m}}{E_{C\_Mital}}-\alpha_{m}{V_{p}}^{0.67}\frac{E_{m}}{E_{C\_Mital}}$$

(8) $$\tilde{\alpha }=\alpha_{m}-{V_{p}}^{0.33}(\alpha_{m}-\alpha_{p})$$

(9) $${\tilde{E}}_{p}=\frac{E_{m}}{1-{V_{p}}^{0.33}(1-\frac{E_{m}}{E_{p}})}$$

(10) $$\kappa_{C\_Mital}=\frac{{V_{p}}^{0.67}\kappa_{m}}{1-{V_{p}}^{0.33}(1-\frac{\kappa_{m}}{\kappa_{p}})}+\left(1-{V_{p}}^{0.67}\right)\kappa_{m}$$

Moreover, Guth suggested a generalized micromechanics model for analyzing particle-reinforced composites based on experimental data on carbon particles immersed in a fluid medium by incorporating empirical variables to account for sphere interactions. The model suggested by Einstein and Guth is extremely effective for composites with low particle densities [42,43]. Conventional empirical formulas for the elastic and shear moduli were used to calculate the thermal expansion coefficient and heat transfer rate. The following equations represent the micromechanics model:

(11) $$E_{C\_Guth}=E_{m}(1+2.5V_{p}+14.1{V_{p}}^{2})$$

(12) $$G_{C\_Guth}=G_{m}(1+2.5V_{p}+14.1{V_{p}}^{2})$$

Halpin and Tsai developed a semi-empirical method for predicting the properties of composites. The Halpin–Tsai model, which involves a mechanism for sensitively interpolating between the upper and lower bounds of composite attributes, predicts the elasticity of a composite based on the shape and orientation of the fillers as well as the elastic characteristics of the fillers and matrix [44,45,46,47,48]. This model employs a reinforcement factor (*ξ*) derived from experiments—considering the shape, location, and loading conditions of the fibers—and helps forecast the composite properties. If the additive is spherical or has a circular cross-section, the reinforcement factor is 2. Equations (13)–(18) [49] represent the Halpin–Tsai model.

(13) $$E_{C\_HT}=E_{m}\frac{1+\xi \eta V_{p}}{1-\eta V_{p}},$$

(14) $$\eta_{E\_HT}=\frac{\frac{E_{p}}{E_{m}}-1}{\frac{E_{p}}{E_{m}}+\xi }$$

(15) $$G_{C\_HT}=G_{m}\frac{1+\xi \eta V_{p}}{1-\eta V_{p}},$$

(16) $$\eta_{G\_HT}=\frac{\frac{G_{p}}{G_{m}}-1}{\frac{G_{p}}{G_{m}}+\xi }$$

(17) $$\kappa_{C\_HT}=\kappa_{m}\frac{1+\xi \eta V_{p}}{1-\eta V_{p}},$$

(18) $$\eta_{\kappa \_HT}=\frac{\frac{\kappa_{p}}{\kappa_{m}}-1}{\frac{\kappa_{p}}{\kappa_{m}}+\xi }$$

Several models have been developed to simulate the CTE of composites. CTE predictions can be derived in general from the ROM. However, ROM-model predictions reveal considerable deviations from observed values [39,50]. Based on the bulk modulus of the filler and matrix, the Kerner model calculates the CTE of a composite. The composite should comprise spherical particles spread throughout the matrix and wetted by a homogeneous layer of the matrix in this model. The composites assumed to be isotropic and homogenous are applicable to the model. The measured CTE of the composite was consistent with the experimental results analyzed by Shin and Lee [51]. Thus, the CTE values estimated for the composite may also be determined to be in good agreement. Furthermore, standard equations, such as Equation (20), can be used to calculate the relationship between *K* and *G* for the matrix and additives.

(19) $$\alpha_{C\_Kerner}=\alpha_{m}V_{m}+\alpha_{p}V_{p}+\left(\alpha_{p}+\alpha_{m}\right)V_{p}V_{m}\left(\frac{\frac{1}{K_{m}}-\frac{1}{K_{p}}}{\frac{V_{m}}{K_{p}}+\frac{V_{p}}{K_{m}}+\frac{3}{4G_{m}}}\right)$$

(20) $$K=\frac{E}{3(3-\frac{E}{G})}$$

## 3. Results

### 3.1. Elastic Modulus

**Figure 2:**
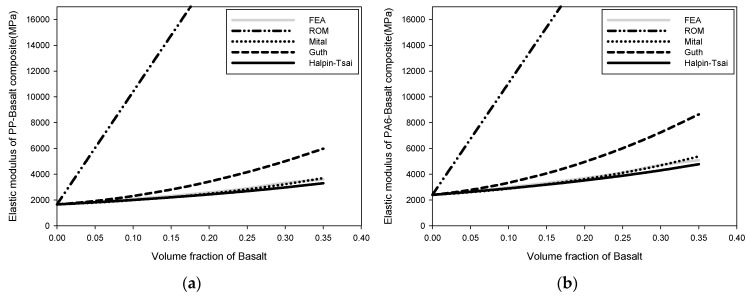

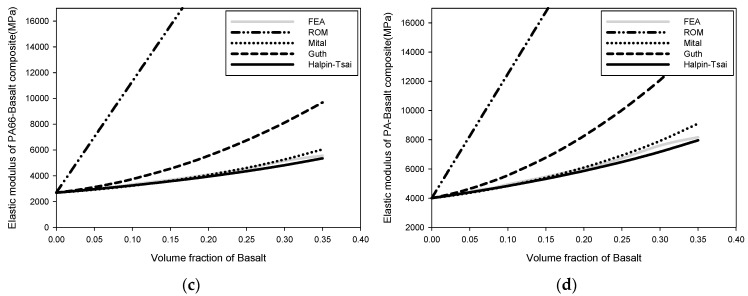
Change in elastic modulus: (**a**) elastic modulus of PP-basalt composite, (**b**) elastic modulus of PA6-basalt composite, (**c**) elastic modulus of PA66-basalt composite, and (**d**) elastic modulus of PA-basalt composite.

The effective properties of composites fabricated by combining basalt powder in PP, PA6, PA66, and PA matrices were calculated in this study. The elastic modulus, shear modulus, thermal expansion coefficient, and heat-transfer rate were considered as the effective properties. As the effective properties of composites vary with the volume fraction of the reinforcements and additives, the volume fraction of basalt powder varied from 2 to 35%, and the changes in the effective properties were investigated. Figure 2 depicts the fluctuation of the elastic modulus of the basalt composite. When the volume percentage of basalt was 35%, the elastic modulus of the PP-basalt composite increased from 1744.1 MPa to 3600.2 MPa, based on the estimation of the change in elastic modulus using the FEA. The PA6-, PA66-, and PA-basalt composites exhibited increased strength from 2518.7 MPa to 5081.8 MPa, 2819.1 MPa to 5557.4 MPa, and 4190.7 MPa to 8168.1 MPa, respectively. The elastic modulus was shown to increase as the volume fraction of basalt increased, owing to the high modulus of elasticity of basalt. Additionally, if the basalt had a good surface bond with the matrix, it suppressed composite deformation and had a high modulus. The FEA results and Mital model likewise exhibited the highest convergence. Moreover, the effective properties of the Halpin–Tsai model converged with the FEA results within a 10 ± 2% error. The ROM approach is determined by linearly substituting the material properties according to the volume fraction of each material; therefore, the error with other micromechanics models is significant. Good convergence with the FEA was observed in the case of the Guth model at basalt-volume fractions < 10%, but it did not improve as the volume fraction increased. The PP-basalt composite showed the lowest elastic modulus of the matrices because PP has the lowest elastic modulus. The elastic modulus of the PA-basalt composite was the highest.

### 3.2. Shear Modulus

**Figure 3:**
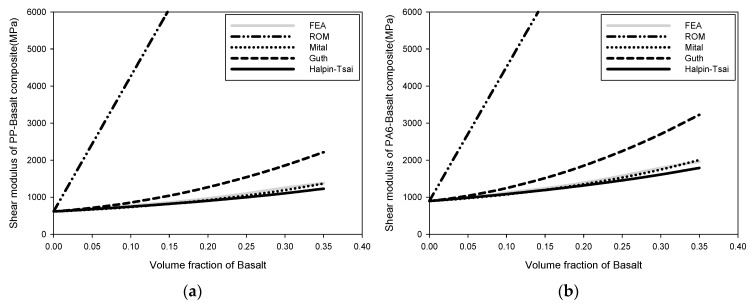

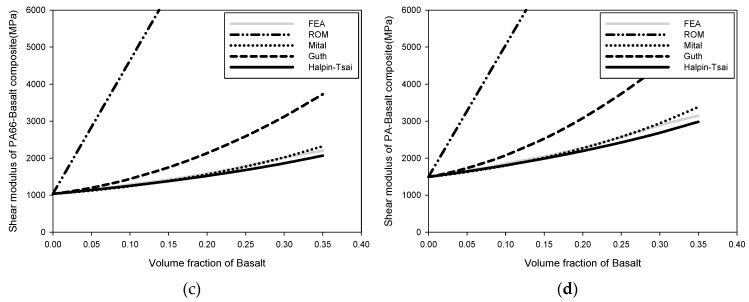
Change in shear modulus: (**a**) shear modulus of PP-basalt composite, (**b**) shear modulus of PA6-basalt composite, (**c**) shear modulus of PA66-basalt composite, and (**d**) shear modulus of PA-basalt composite.

Figure 3 depicts the change in the shear modulus of the composite. The change in shear modulus with the addition of basalt powder followed a similar pattern to the change in elastic modulus. The shear modulus of the PP-basalt composite increased from 646.51 MPa to 1379.5 MPa when the basalt volume percentage was 35%, according to the FEA data. The shear modulus of the PA66-basalt composite increased from 1085.2 MPa to 2201.7 MPa, whereas that of the PA-basalt composite increased from 4190.7 MPa to 8168.1 MPa. The shear modulus was observed to increase as the volume fraction of basalt increased. The following table compares the findings of the micromechanics model with the results of the FEA model based on the volume percentage of basalt. The best convergence was observed between the FEA results and the Mital and Halpin–Tsai models. The convergence impact of the Guth model is good for low-volume fractions. The ROM model exhibited a considerable change in its number of steps, and the FEA error was significant.

### 3.3. Coefficient of Thermal Expansion

**Figure 4:**
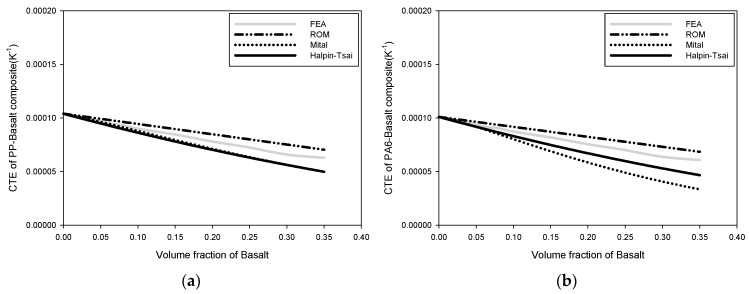

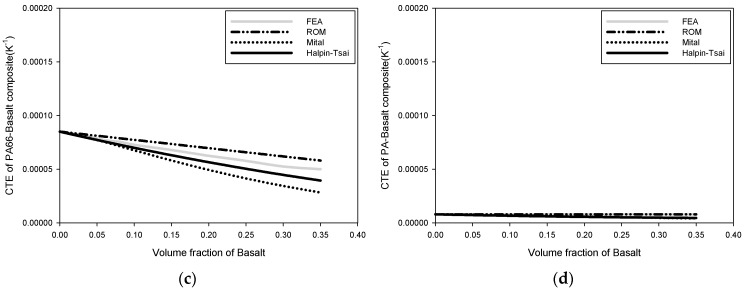
Change in thermal expansion coefficient: (**a**) CTE of PP-basalt composite, (**b**) CTE of PA6-basalt composite, (**c**) CTE of PA66-basalt composite, and (**d**) CTE of PA-basalt composite.

Figure 4 depicts the fluctuation of the thermal expansion coefficient with the volume fraction of reinforcement in the composite. When the thermal expansion coefficient was predicted using the FEA, it decreased as the reinforcing agent increased. The ROM approach and the Halpin–Tsai method of determining the thermal expansion coefficient produced findings that were the most similar to those of the FEA in the case of micromechanics. As the thermal expansion coefficient of each material was similar in PA-basalt composites, the change in the thermal expansion coefficient was not significant, even if the composite was fabricated.

### 3.4. Thermal Conductivity

**Figure 5:**
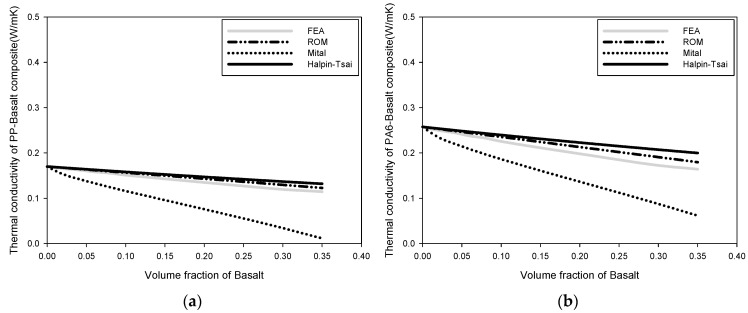

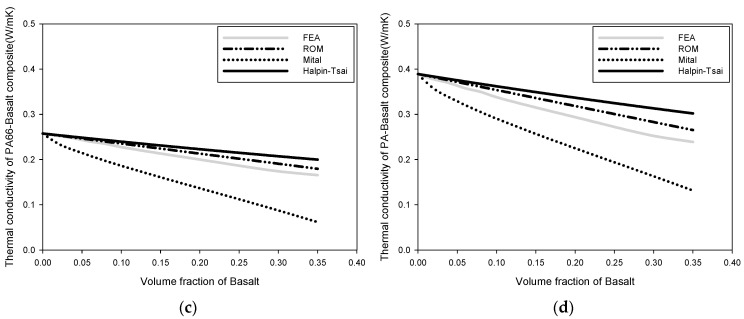
Change in heat-transfer rate: (**a**) thermal conductivity of PP-basalt composite, (**b**) thermal conductivity of PA66-basalt composite, (**c**) thermal conductivity of PA66-basalt composite, and (**d**) thermal conductivity of PA-basalt composite.

Figure 5 depicts a comparison of the heat-transfer rates. As the volume proportion of the reinforcement increased, the change in the heat-transfer rate decreased. The micromechanics and FEA results were compared to calculate this change in the heat-transfer rate. The FEA, the ROM, and the Halpin–Tsai model produced comparable results, whereas the Mital method produced considerably different results. The error in the Mital method was observed to increase as the volume fraction of the reinforcement increased.

## 4. Discussion

In this study, the effective properties of composites containing basalt powder were investigated using FEA and micromechanics models. Composite matrices—PP, PA6, PA66, and PA, using basalt powder as a reinforcing agent—were employed to model an isotropic composite. The basic ROM model includes two models. The first model, proposed by Voigt, is commonly used to compute the modulus of elasticity in the fiber direction, whereas the second model, proposed by Rouss, can be used to estimate the modulus of elasticity in the transverse direction. Both methods share the assumption that the composite microstructure is evaluated as a simple block using volume fractions. However, most composites have complex geometries that are not simply two blocks connected together. The composites employed in this study are powder blends; thus, the basalt powder is distributed randomly inside the composite. Therefore, the error range for assessing the effective properties of composites using ROM is considerable. As a result, many experiments have been conducted to overcome this error range by substituting experimental results or altering the ROM model through correction factors [52,53,54]. The results of this study show that the elastic and shear moduli utilizing ROM increase linearly, which differs from the results of FEA and other micromechanics methods. In contrast, the thermal expansion coefficient and heat-transfer rate results obtained utilizing the ROM approach were identical to those of FEA.

Among other micromechanics models, Mital and Goldberg conducted a study to describe the micromechanics equations for particulate-reinforced composites and predict their effective thermal and mechanical properties as well as the constituent microstresses resulting from applied thermal and mechanical loadings. They presented a material degradation model, characterized the material, and derived effective prediction equations using simulations [42]. The Mital model showed good convergence for the computation of the elastic and shear moduli in this study, with the findings comparable to those of FEA. However, when analyzing heat-transfer characteristics, the error of the Mital model is the greatest, indicating that it is unsuitable for computing composite heat-transfer characteristics.

The Guth model is the diluted estimate of Einstein’s model, which derived the increase in viscosity caused by a suspension of spherical particles in a viscous fluid. The same approach was subsequently applied by Smallwood to predict the small strain of Young’s modulus of particle-filled solids. However, this estimate is only effective at low (i.e., diluted) filler concentrations [55]. The effective properties, i.e., elastic and shear moduli, of basalt tend to converge with the FEA results up to a powder volume fraction of 10% based on these model characteristics. However, when the volume fraction exceeded 15%, the convergence was poor, and the error increased.

The Halpin–Tsai model has been used to study the effective properties of composites by configuring parameters depending on the particle aspect ratio [44,47] and can accurately predict the elastic modulus, shear modulus, thermal expansion coefficient, and heat-transfer parameters.

Finally, the optimal conditions for manufacturing a polymer-based composite of basalt powder can be shown in Table 2. The analytical results and micromechanics analysis showed that the best physical properties were obtained when the volume fraction of basalt was 35%, i.e., the maximum.

An analysis of the effective properties of composites showed that composites could be successfully fabricated with PA as a matrix to make basalt composites with high tensile elasticity and shear-deformation resistance. Furthermore, because the elastic moduli of PA and basalt are similar, the damage caused by delamination and the thermal expansion coefficient discrepancies produced by temperature fluctuations in composites may be reduced. In addition, creating composites with heat-dissipation qualities is feasible because of the high thermal conductivity of PA. Thus, a composite material with excellent physical qualities may be obtained by preparing an isotropic composite material by mixing basalt powder with PA. Therefore, if the hydrogen bonding and surface bonding between the basalt powder and the polymer matrix can be maintained well, it is expected that the basalt fiber can play the role of reinforcing agent inside the polymer.

## 5. Conclusions

We conducted a preliminary analysis of the effective properties of manufactured bas-alt particulate–polymer composites. The effective properties of the isotropic composites fabricated using polymer materials such as PP, PA6, PA66, and PA, with a basalt spherical model used as a reinforcing agent, were investigated. To calculate the effective properties, the elastic modulus, shear modulus, thermal expansion coefficient, and thermal conductivity of basalt particles were investigated using a micromechanics model and FEA. Furthermore, the results of the micromechanics model were compared to those of FEA, appropriate for the development of particulate composites. If it is possible to produce composite materials due to the good binding of basalt powder and polymer, the following conclusions can be reached. As the volume fraction of basalt particles increases, mechanical properties such as the elastic modulus and shear modulus are improved. On the other hand, as the volume fraction of basalt powder increases, the coefficient of thermal expansion and thermal conductivity decrease. Depending on the type of polymer used in the composite, the results are as follows. PA-based composites had excellent physical qualities when designed as isotropic composites with basalt powder. Furthermore, when micromechanics models were employed to study the effective properties of the composites, the Halpin–Tsai model predicted the effective properties well. In the future, further research will be required to develop and use composite materials based on the examination and assessment of the effective properties of particulate composites to build composite materials that can be used in injection-molding processes.

## Figures and Tables

**Table 1:** Properties of materials [29,30,31,32,33,34].

Properties	Units	PP	PA	PA6	PA66	Basalt
Density	g/cm^3^	0.90	1.10	1.13	1.140	2.75
Thermal expansion coefficient	1/K	104 × 10^−6^	8 × 10^−6^	1.01 × 10^−4^	85 × 10^−6^	8 × 10^−6^
Elastic modulus	MPa	1660	4000	2400	2690	89,000
Poisson’s ratio		0.35	0.34	0.34	0.30	0.20
Shear modulus	MPa	614.81	1492.54	895.52	1034.62	37,083.33
Thermal conductivity	W/mK	0.17	0.39	0.26	0.26	0.035

**Table 2:** Predicting the effective properties of composites with FEA.

(Volume Fraction) Composite	Elastic Modulus (MPa)	Shear Modulus (MPa)	CTE (μm/K^−1^)	Thermal Conductivity (W/mk)
PP(35)Basalt	3600.0	1379.5	62.91 × 10^−6^	0.115
PA6(35)Basalt	5081.8	1960.3	60.86 × 10^−6^	0.164
PA66(35)Basalt	5557.4	2201.7	50.02 × 10^−6^	0.166
PA(35)Basalt	8168.1	3149.0	8.00 × 10^−6^	0.239
